# *N*-Glycosylation Site in the Middle Region Is Involved in the Sperm-Binding Activity of Bovine Zona Pellucida Glycoproteins ZP3 and ZP4

**DOI:** 10.3390/biom13111636

**Published:** 2023-11-10

**Authors:** Kamila Dilimulati, Zhang Yulin, Fabiana Lica Imai, Naoto Yonezawa

**Affiliations:** 1 Department of Chemistry, Graduate School of Science, Chiba University, Chiba 263-8522, Japan; kamila221@hotmail.com (K.D.); licaimai@hotmail.com (F.L.I.); 2 Department of Quantum Life Science, Graduate School of Science and Engineering, Chiba University, Chiba 263-8522, Japan; zhangylecnu@outlook.com

**Keywords:** zona pellucida, sperm-binding site, fertilization, hinge region, *N*-glycosylation

## Abstract

Mammalian fertilization is a species-selective event that involves a series of interactions between sperm proteins and the oocyte’s zona pellucida (ZP) glycoproteins. Bovine ZP consists of three glycoproteins: bZP2, bZP3, and bZP4. In our previous study, we demonstrated that bovine sperm binds to plastic wells coated with recombinant bZP4 and identified that the *N*-terminal domain and the middle region of bZP4 are critical for sperm-binding activity. Here, we investigated the sperm-binding site in the middle region (residues 290 to 340) of bZP4, which includes the hinge region. We showed that bovine sperm binds to bZP4’s middle region in a species-selective manner. We mapped the function of bZP4’s middle region to its *N*-glycosylation site at Asn-314 using several recombinant mutated proteins. Moreover, we showed that mutations of the *N*-glycosylation sites at Asn-314 close to the hinge region and Asn-146 of the hinge region of bZP4 and bZP3, respectively, reduced the sperm-binding activity of the complex of the bZP3 (from 32 to 178) and bZP4 (from 136 to 464) fragments. Together, these results suggest that ZP’s middle regions of bZP3 and bZP4 form one of the sperm-binding sites of bovine ZP.

## 1. Introduction

**Figure 1:**
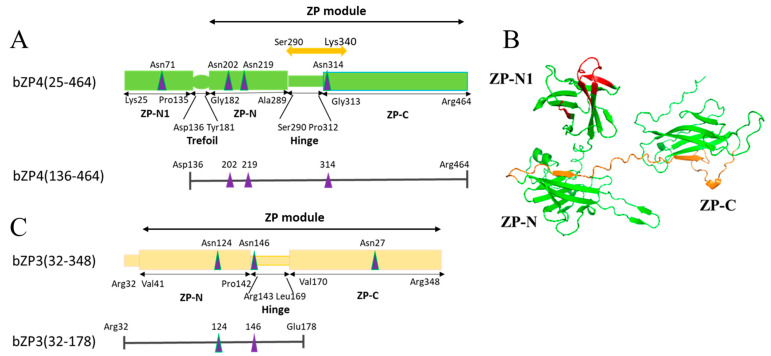
Domain architectures of bZP4 and bZP3 polypeptides. (**A**) Domain architecture of mature bZP4 (25–464) and schematic representation of truncated bZP4 (136–464) fragment. The orange two-headed arrow shows the bZP4 (290–340) region examined in this study. The inverted tripods mark potential *N*-glycosylation sites. *O*-glycosylation sites are not shown. (**B**) A predicted structural model of bZP4 (25–464). This is a top-ranked model made using AlphaFold2. The previously proposed three sites involved in sperm binding on bZP4 (25–136) are shown in red in the ZP-N1 domain. The previously identified sperm-binding region from 290 to 340 on bZP4 is shown in orange extending from ZP-N domain to ZP-C domain. This orange region corresponds to the two-headed orange arrow shown in (**A**). (**C**) Domain architecture of mature bZP3 (32–348) and schematic representation of truncated bZP3 (32–178). The inverted tripods mark potential *N*-glycosylation sites. *O*-glycosylation sites are not shown.

Mammalian oocytes are enveloped by the zona pellucida (ZP), a specialized extracellular coat, which is involved in oogenesis and species-selective sperm recognition. It also participates in blocking polyspermy and protecting the developing embryo [1,2,3]. The ZP of most mammals, including the human ZP, contains four glycoproteins (ZP1–4) [4], whereas mice (m) ZP, porcine (p) ZP, and bovine (b) ZP possess only three (mZP1–3, pZP2–4, and bZP2–4, respectively) [5,6]. All ZP proteins contain a ZP module, necessary for the formation of ZP filaments, which consists of two structurally related immunoglobulin (Ig)-like domains (ZP-N and ZP-C) that are connected by a short, flexible hinge region (see Figure 1A,C) [7,8,9]. Despite the structural similarity in the amino acid sequences, ZP proteins are highly heterogeneous due to their post-translational modifications, including glycosylation of *N* (asparagine)- and *O* (serine/threonine)-linked carbohydrates, whose compositions, such as sialylation and sulfation, vary across several mammalian species [3].

Studies have shown the role of ZP proteins in sperm binding in several mammalian species. In mice and humans, sperm binds to the *N*-terminal domain of ZP2 in a species-selective manner [10]. Its polypeptide moiety, instead of the carbohydrate moiety, is responsible for this binding [11]. Though only in vitro studies have been reported, evidence suggests that different ZP proteins are involved in the sperm-binding processes in pigs and bovines [3,12,13]. Nevertheless, ZP4 predominantly binds to the sperm in both pigs and bovines [12,13], indicating that the mechanisms of sperm–ZP interaction are somewhat conserved between these species. The carbohydrate chains on the porcine and bovine ZP glycoproteins contribute to sperm–ZP binding [14]. Specifically, the non-reducing terminal β-galactosyl (Gal) residues of the complex-type *N*-linked chains are involved in porcine sperm binding [15], and the non-reducing terminal α-mannosyl (Man) residues of the high-mannose-type chain Man_5_GlcNAc_2_ are implicated in bovine sperm binding [16].

Competitive inhibition assays have shown that solutions containing bovine bZP3/bZP4 heterocomplex can inhibit sperm–ZP binding, whereas neither bZP3 nor bZP4 alone could inhibit this binding [17]. This suggests that the bZP3/bZP4 heterocomplex formation is necessary for in vitro sperm-binding activity. Recently, using ZP proteins fixed to plastic wells, we demonstrated that bovine sperm binds to bZP4 but not to bZP3, and that two regions of bZP4 are involved in sperm binding [13]. The first region, ranging from Lys-25 to Asp-136, nearly corresponds to the *N*-terminal ZP-N1 domain (Figure 1A,B). The second region, extending from Ser-290 to Lys-340, comprises the flexible hinge region and the *N*-terminus of the ZP-C domain (Figure 1A,B) [3,18]. Bovine sperm binds to bZP4 (25–136) in a species-selective manner without requiring *N*-glycosylation [19]. Additionally, we identified three sites involved in sperm binding on bZP4 (25–136) using chimeric bovine/porcine and bovine/human ZP4 recombinant proteins (Figure 1B) [19]. However, the role of the bZP4 (290–340) region in sperm–ZP binding remains to be determined.

Notably, the *N*-terminal fragment of bZP3 (Arg-32 to Glu-178), which includes the hinge region (Figure 1C), interacts with bZP4. Competitive inhibition assay results showed that this complex inhibits bovine sperm–ZP binding [20]. Furthermore, when the *N*-glycosylated Asn-146 from the bZP3 fragment’s hinge region was mutated to Asp, the inhibitory activity was reduced, even though this mutation did not diminish the interaction between the bZP3 fragment and bZP4 [20]. This result suggested the significance of the *N*-glycosylation site on bZP3’s hinge region in sperm recognition by the bZP3/bZP4 complex.

To understand the role of the bZP4 middle region (290–340) in sperm-binding activity, we conducted an in vitro sperm–ZP protein-binding assay using recombinant bZP4 mutated proteins.

## 2. Materials and Methods

### 2.1. Construction of Recombinant Baculovirus Transfer Plasmids for ZP Proteins

We prepared pBACgus6 plasmids encoding several mutant sequences for the residues 290 to 340 of bZP4 (136–464) using polymerase chain reaction (PCR) with the PrimeSTAR Mutagenesis Basal Kit (Takara, Kyoto, Japan). We used the pBACgus6 plasmid encoding the *N*-terminally His-tagged and S-tagged bZP4 fragment from Asp-136 to Arg-464 with the translation initiation Met residues numbered 1 (bZP4 (136–464)) [17] as the first-PCR template. PCR primers were commercially synthesized by Eurofins Genomics (Tokyo, Japan). The first PCR involved mutating the Thr-332 of bZP4 (136–464) to Asn at 333 of pZP4 using the primers bZP4T332N sense (5′-TACTACAACGCTAGTGACTACCCAGTG-3′) and bZP4T332N antisense (5′-ACTAGCGTTGTAGTAGGAGCGATAGC-3′) to prepare pBACgus6 encoding bZP4 (136–464)/pZP4 (333). Using the pBACgus6 encoding bZP4 (136–464)/pZP4 (333) as a template for the second PCR, we created two mutations: Arg-328 to Gly at 329 of pZP4 and Lys-325 to Glu at 326 of pZP4 were added to bZP4 (136–464)/pZP4 (333). The primers bZP4R328GK325E sense (5′-ATGAACGCTATGGCTCCTACTACAACGCTA-3′) and bZP4R328GK325E antisense (5′-AGCCATAGCGTTCATCTTTGGCAATCTTAAG-3′) were used to give the pBACgus6 plasmid encoding bZP4 (136–464)/pZP4 (333, 326–329). For the third PCR, we used the pBACgus6 plasmid encoding bZP4 (136–464)/pZP4 (333, 326–329) as a template and mutated Lys-320 to Gln at 321 of pZP4 using the primers bZP4K320Q sense (5′-GGAACTTCAGATTGCCAAAGATGAAC-3′) and bZP4K320Q antisense (5′-GCAATCTGAAGTTCCAGAGTGAGGTT-3′) to prepare pBACgus6 encoding bZP4 (136–464)/pZP4 (333, 326–329, 321). For the fourth PCR, using the pBACgus6 plasmid encoding bZP4 (136–464)/pZP4 (333, 326–329, 321) as a template, we created two mutations: Asn-314 to Pro at 315 of pZP4 and Gln-311 to His at 312 of pZP4 using the primers bZP4N314PQ311H sense #1 (5′-CATCCTGGACCACTCACTCTGGAACTTCAG-3′) and bZP4N314PQ311H antisense (5′-GAGTGGTCCAGGATGGGTCTCAGGAAGGGGT-3′) to give pBACgus6 encoding bZP4 (136–464)/pZP4 (333, 326–329, 321, 312–315). For the fifth PCR, the pBACgus6 encoding bZP4 (136–464)/pZP4 (333, 326–329, 321, 312–315) was used as a template to create two mutations—Leu-302 to Phe at 303 of pZP4 and Val-298 to Ile at 299 of pZP4—using the primers bZP4L302FV298I sense (5′-ATATCCAGGTTTTTACTCTCCCACCACCCCT-3′) and bZP4L302FV298I antisense (5′-TAAAAACCTGGATATTAACTGGGAGAGCACT-3′) to give the pBACgus6 encoding bZP4 (136–464)/pZP4 (333, 326–329, 321, 312–315, 299–303).

The pBACgus6 plasmid encoding bZP4 (136–464)/pZP4 (312–315) was prepared using the PrimeSTAR Mutagenesis Basal Kit (Takara) by PCR using the primers bZP4N314PQ311H sense #2 (5′-CATCCTGGACCACTCACTCTGGAACTTAAG-3′) and bZP4N314PQ311H antisense (shown above) using the pBACgus6 encoding bZP4 (136–464) as a template. The pBACgus6 plasmid encoding bZP4 (136–464)/pZP4 (312–315, 299–303) was prepared using the PrimeSTAR Mutagenesis Basal Kit (Takara) and the bZP4L302FV298I sense and antisense primers (shown above) with the pBACgus6 plasmid encoding bZP4 (136–464)/pZP4 (312–315) as a template.

The pBACgus6 plasmids encoding bZP4 (136–464) with a single amino acid mutation of Gln-311 to His (bZP4 (136–464) Q311H), bZP4 (136–464) with mutation of Asn-314 to Pro (bZP4 (136–464) N314P), and bZP4 (136–464) with mutation of Asn-314 to Asp (bZP4 (136–464) N314D) were prepared using PCR with the PrimeSTAR Mutagenesis Basal Kit (Takara) and the primers bZP4Q311H sense (5′-AGACCCATCCTGGAAACCTCACTCTG-3′) and bZP4Q311H antisense (5′-TTCCAGGATGGGTCTCAGGAAGGGGT-3′) for bZP4 (136–464) Q311H, the primers bZP4N314P sense (5′-CCTGGACCACTCACTCTGGAACTTAAG-3′) and bZP4N314P antisense (5′-AGTGAGTGGTCCAGGCTGGGTCTCAGG-3′) for bZP4 (136–464) N314P, the primers bZP4N314D sense (5′-GCCTGGAGACCTCACTCTGGAACTTA-3′) and bZP4N314D antisense (5′-GTGAGGTCTCCAGGCTGGGTCTCAGG-3′) for bZP4 (136–464) N314D, and the pBACgus6 plasmid encoding bZP4 (136–464) as a template.

The pBACgus6 plasmid encoding *N*-terminal FLAG-tagged and S-tagged bZP4 (136–464) was prepared by deleting the DNA fragment encoding the bZP4 ZP-N1 domain using the PrimeSTAR Mutagenesis Basal Kit (Takara) and the primers bZP4ZPN1del sense (5′-CCCGGGCGATGTCCCAAATGCTGGC-3′) and bZP4ZPN1del antisense (5′-GGGACATCGCCCGGGCTCTTGTCGTC-3′) with the pBACgus6 encoding FLAG- and S-tagged bZP4 [17] as a template. The Asn-314 on bZP4 (136–464) was mutated to Asp using the PrimeSTAR Mutagenesis Basal Kit (Takara) and the bZP4N314D sense and antisense primers to obtain the pBACgus6 plasmid encoding FLAG- and S-tagged bZP4 (136–464) N314D.

The DNA sequences of the constructed plasmids were confirmed using a commercial DNA sequencing service (Eurofins Genomics, Tokyo, Japan).

### 2.2. Expression of Recombinant ZP Proteins

The recombinant baculoviruses for bZP4, bZP4 (136–464), pZP4 (137–466), bZP3 (32–178), and bZP3 (32–178) N146D, all of which are *N*-terminally His- and S-tagged, were reported previously [17,20,21]. New recombinant baculoviruses were prepared based on a previously reported procedure [13]. Sf9 cells were routinely propagated in Sf-900II serum-free medium (Invitrogen, Carlsbad, CA, USA) and transfected with each baculovirus transfer plasmid construct along with flashBAC DNA (Oxford Expression Technologies, Oxford, UK), following the manufacturer’s protocol. The Sf9 cells were then infected with each recombinant virus. The expression and secretion of each recombinant protein into the culture supernatant were verified, as previously reported [17,19]. All recombinant ZP proteins were expressed as secretory proteins using the signal peptide from pBACgus6.

### 2.3. Purification of Recombinant ZP Proteins from Culture Supernatants

The recombinant ZP proteins were purified as reported previously [17,19]. Briefly, for large-scale protein expression, either an individual or a mixture of corresponding recombinant virus(es) was used to infect 200 mL of Sf9 cells (1 × 10^6^ cells/mL). These suspensions were cultured for 48 h at 27 °C. Each *N*-terminal His-tagged recombinant protein or complex was purified using TALON metal affinity resin^®^ (Clontech, Mountain View, CA, USA). The protein concentrations were determined at an absorbance of 280 nm using the absorbance value of 1 mg/mL protein, which was calculated based on their amino acid compositions (https://web.expasy.org/protparam/ (accessed on 18 April 2023)).

### 2.4. SDS-PAGE

SDS-PAGE was performed on 11 or 12.5% (*w*/*v*) separating gels under reducing conditions according to the Laemmli method [22]. The gels were silver-stained, and the standard proteins with a broad molecular mass range (Takara) were used to estimate the apparent protein molecular masses.

### 2.5. Adsorption of Recombinant ZP Proteins to Plastic Wells

We determined the amount of recombinant ZP protein sufficient for saturated adsorption into plastic wells by adding 50 μL of protein solution at varying concentrations in TALON elution buffer (20 mM Tris-HCl, pH 7.9, 150 mM imidazole, and 150 mM NaCl) to a 96-well plate (Nalge Nunc, Rochester, NY, USA) and incubating overnight at 4 °C [13,19]. We included a control well without proteins or with bZP4. The wells were then rinsed with phosphate-buffered saline (PBS: 28.8 mM Na_2_HPO_4_/11.2 mM NaH_2_PO_4_, pH 7.4, 52 mM NaCl) and blocked with 3% bovine serum albumin (BSA) in Tris-buffered saline (TBS: 20 mM Tris-HCl, pH 7.5, 150 mM NaCl) at room temperature for 1 h. After washing with TBS, each well was incubated with an anti-His-tag monoclonal antibody (clone No.: 9C11, Wako, Kyoto, Japan) alone (1/3000 with TBS containing 1% BSA), or with a mixture of anti-His-tag and anti-FLAG-tag monoclonal antibodies (clone No.: 1E6, Wako) (1/3000 with TBS containing 1% BSA) for 1 h at room temperature. The wells were washed thrice with TBS containing 0.05% Tween 20 (T-TBS) and incubated with horseradish peroxidase (HRP)-conjugated rabbit anti-mouse IgG antibody (Wako) (1/1000 in TBS containing 1% BSA). After washing thrice with T-TBS, the wells were incubated with 2,2’-Azinobis (3-ethylbenzothiazolin-6-sulfonic Acid) (ABTS; Roche, Mannheim, Germany) as the HRP substrate. The absorbance was measured at 405 nm using a plate reader (TECAN, Grödig, Austria) after incubating for 1 h at room temperature.

### 2.6. Sperm Binding to Recombinant ZP Proteins Adsorbed to Plastic Wells [13,19]

**Figure 2:**
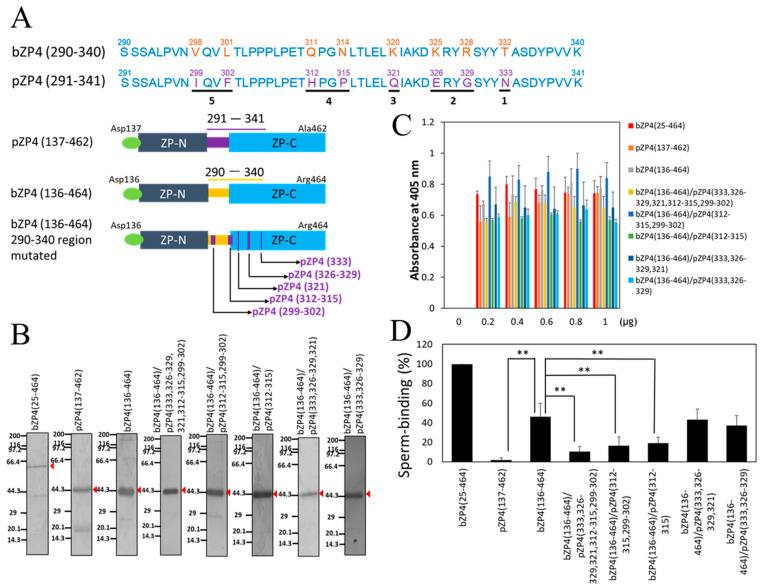
Sperm-binding sites in the middle region of bZP4. (**A**) Comparison of the amino acid sequences of bZP4 (290–340) and its corresponding pZP4 (291–341) counterpart and schematic diagrams of pZP4 (137–462), bZP4 (136–464), and bZP4 (136–464) mutant proteins. The non-identical amino acid residues between bovine and porcine sequences are highlighted in yellow and purple, respectively. Amino acids are numbered considering translational initiation at Met as 1. The schematic diagram of bZP4 (136–464) mutant proteins shows the pZP4 (333) (region 1), pZP4 (326–329) (region 2), pZP4 (321) (region 3), pZP4 (312–315) (region 4), and pZP4 (299–302) (region 5) that were replaced in the corresponding bovine sequences. (**B**) SDS-PAGE of bZP4 (25–464), bZP4 (136–464), pZP4 (137–462), and bZP4 (136–464) fragments with the indicated sites mutated with corresponding porcine sequences. Red arrowheads indicate protein bands of bZP4 (25–464) and bZP4 (136–464) and its mutants. Gels were silver-stained. Molecular mass standards (kDa) are indicated on the left side of each panel. SDS-PAGE original images can be found in Supplementary Materials. (**C**) Adsorption of each recombinant ZP4 protein to plastic wells shown in (**B**). The amount of each recombinant ZP4 protein added to a plastic well is indicated under each group of bars. The amounts of protein necessary for adsorption saturation were examined by detecting the adsorbed proteins with an anti-His tag antibody. The experiment was performed thrice, and the average ± standard deviation (SD) of absorbance at 405 nm is shown. (**D**) Sperm-binding activity of the recombinant ZP4 proteins. Plastic wells were coated with each protein (0.8 μg) shown in (**C**). The number of sperm bound to the wells coated with bZP4 (25–464) varied from 29 to 58 but was designated as 100% for each experiment. Assays were repeated five times. Data are presented as the mean ± SD, with statistical significance between two bars indicated as *p* < 0.01 (**) on each line connecting the two bars.

**Figure 3:**
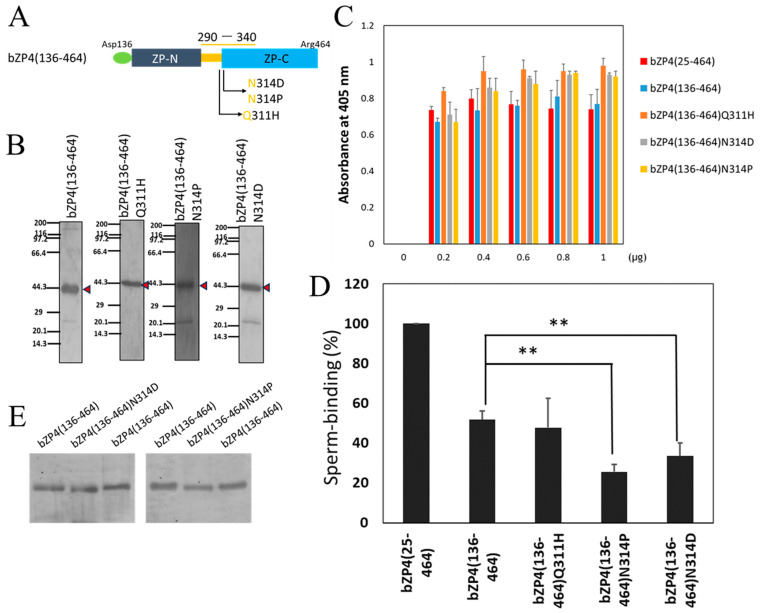
The *N*-glycosylation site on bZP4 (290–340) is involved in the sperm-binding activity of bZP4. (**A**) Schematic of bZP4 (136–464) with the mutation of Gln-311 and Asn-314 sites. (**B**) SDS-PAGE of bZP4 (136–464) Q311H, bZP4 (136–464) N314P, and bZP4 (136–464) N314D mutant proteins. Red arrowheads indicate protein bands of bZP4 (136–464) and its mutants. Gels were silver-stained. Molecular mass standards (kDa) are indicated on the left side of each panel. SDS-PAGE original images can be found in Supplementary Materials. (**C**) Adsorption of each bZP4 mutant protein to plastic wells shown above. The amount of each bZP4 protein added to a plastic well is indicated under each group of bars. The amounts of proteins necessary for adsorption saturation were examined by detecting the adsorbed proteins with an anti-His tag antibody. The experiment was performed thrice, and the average ± standard deviation (SD) of absorbance at 405 nm is shown. (**D**) Sperm-binding activity of each bZP4 protein shown above. Plastic wells were coated with each bZP4 protein (0.8 μg) indicated in the graph. The number of sperm bound to the wells coated with bZP4 (25–464) varied from 23 to 42 but was designated as 100% for each experiment. Assays were repeated at least four times. Data are presented as the mean ± SD, with statistical significance between two bars indicated as *p* < 0.01 (**) on each line connecting the two bars. (**E**) Comparisons of mobilities on SDS-PAGE gels between bZP4 (136–464) and its *N*-glycosylation site mutants. Left panel: mobilities were compared between bZP4 (136–464), left and right lanes, and bZP4 (136–464) N314D, middle lane. Right panel: mobilities were compared between bZP4 (136–464), left and right lanes, and bZP4 (136–464) N314P, middle lane. Gels were silver-stained. Only the parts around the protein bands are shown. SDS-PAGE original images can be found in Supplementary Materials.

**Figure 4:**
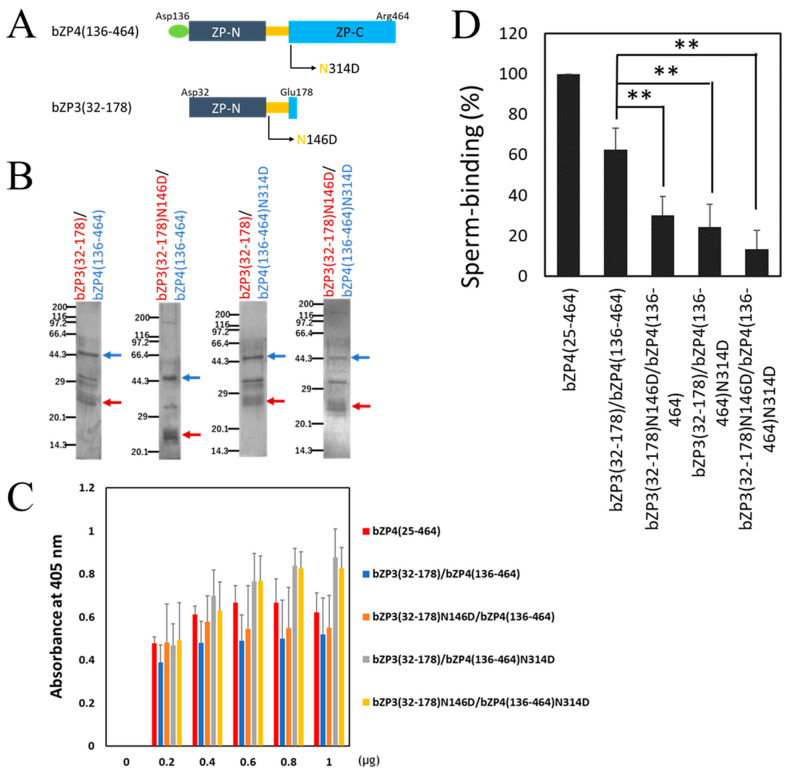
*N*-glycosylation sites in the middle region of both bZP3 and bZP4 are involved in sperm-binding activity. (**A**) Schematic diagrams of bZP4 (136–464) with mutation of Asn-314 site and bZP3 (32–178) with mutation of Asn-146 site. The Asn-314 and Asn-146 were mutated to Asp (N314D and N146D, respectively). (**B**) SDS-PAGE images of bZP3 (32–178)/bZP4 (136–464), bZP3 (32–178) N146D/bZP4 (136–464), bZP3 (32–178)/bZP4 (136–464) N314D, and bZP3 (32–178) N146D/bZP4 (136–464) N314D. Blue arrows indicate protein bands of bZP4 (136–464) and bZP4 (136–464) N314D. Red arrows indicate protein bands of bZP3 (32–178) and bZP3 (32–178) N146D. Gels were silver-stained. Molecular mass standards (kDa) are indicated on the left side of each panel. SDS-PAGE original images can be found in Supplementary Materials. (**C**) Adsorption of each complex protein to plastic wells shown above. The amount of each complex protein added to a plastic well is indicated under each group of bars. The amounts of proteins necessary for adsorption saturation were examined by detecting the adsorbed proteins with a mixture of anti-His tag and anti-FLAG tag antibodies. The experiment was performed thrice, and the average ± standard deviation (SD) of absorbance at 405 nm is shown. (**D**) Sperm-binding activity of each protein complex shown above. Plastic wells were coated with each protein complex (0.8 μg for each protein complex). The number of sperm bound to wells coated with bZP4 (25–464) varied from 21 to 39 but was designated as 100% for each experiment. Assays were repeated at least four times. Data are presented as the mean ± SD, with statistical significance between two bars indicated as *p* < 0.01 (**) on each line connecting the two bars.

Specific amounts of the proteins were added to a 96-well plate (Nalge Nunc) and incubated overnight at 4 °C. Only 50 μL of TALON elution buffer was adsorbed as the negative control. After discarding the solution, the wells were washed with PBS and blocked with 3% BSA in TBS at 38.5 °C for 2 h. Frozen Holstein bull sperm straws, purchased from Animal Genetics Japan Co., Ltd. (Matsuzaka, Japan), were used for artificial insemination. Frozen bovine sperm was thawed, washed twice in pre-warmed (38.5 °C) Brackett and Oliphant (BO) solution without BSA [23], and then capacitated by incubating in BO solution containing BSA for 30 min. The capacitation and subsequent incubations were performed at 38.5 °C under 2% CO_2_. Aliquots (50 μL) containing 4 × 10^5^ capacitated sperm were transferred into the wells, and the plates were incubated for 2 h. After incubation, the wells were washed thrice with BO solution, and 50 μL of 70% glycerol in PBS was added to each well. The sperm bound to the wells was recovered by 20 strokes of vigorous pipetting. Then, the number of sperm in 0.1 μL of suspension was determined using a hemocytometer. The number of sperm bound to the uncoated wells was subtracted from the number of sperm bound to the wells coated with recombinant ZP proteins. The average number of sperm in 0.1 μL of suspension in the 100% sperm-binding control of experiments is provided in the figure legends (Figure 2, Figure 3 and Figure 4).

### 2.7. Statistical Analysis

We used Welch’s *t*-test to determine whether there was a significant difference in sperm count between the two groups. Differences with *p* < 0.05 were considered statistically significant.

### 2.8. Modeling of bZP Proteins

Models of bZP4 (25–464) and heterodimer of bZP3(32–178) and bZP4 (25–464) were generated using the ColabFold v1.5.2-patch: AlphaFold2 website using MMseqs2 (https://colab.research.google.com/github/sokrypton/ColabFold/blob/main/AlphaFold2.ipynb (accessed on 11 October 2023)) with template mode: none [24].

## 3. Results

### 3.1. Identification of Sperm-Binding Sites on bZP4 (290–340)

The mature polypeptide of bZP4, extending from Lys-25 to Arg-464 with translational initiation Met numbered 1, consists of five regions: (1) the *N*-terminal ZP-N-like domain (Lys-25 to Pro-135, ZP-N1), (2) the trefoil domain (Asp-136 to Tyr-181), (3) the ZP-N domain (Gly-182 to Ala-289, ZP-N), (4) the hinge region (Ser-290 to Pro-312), and (5) the ZP-C domain (Gly-313 to Arg-464, ZP-C) (Figure 1A). Previously, we showed that one of the sperm-binding regions of bZP4 is located on bZP4 (290–340), which includes the hinge region and the *N*-terminal region of the ZP-C domain (Figure 1A,B) [13]. The amino acid sequence of bZP4 (290–340) and its corresponding pZP4 (291–341) share 84% sequence identity (Figure 2A). Therefore, considering the lack of conservation between these residues in bovines and pigs, we focused on this region. Analyzing the bZP4 (290–340) fragment on SDS-PAGE is challenging due to the low molecular mass of the fragment. Moreover, the region beside this 290 to 340 fragment on bZP4 (136–464) is not necessary for its sperm-binding activity [13]. Therefore, we selected bZP4 (136–464) for analysis.

We compared the binding of bovine sperm to bZP4 (136–464) and pZP4 (137–466) to understand whether the sperm recognizes the middle region (291–341) of pZP4. His-tagged bZP4 (136–464) and His-tagged pZP4 (137–466) were expressed as secretory proteins using the Sf9-baculovirus system. The recombinant proteins were purified from the culture supernatants with TALON resin utilizing the *N*-terminal His-tag (Figure 2B). There were contaminated protein bands around 20–30 kDa, but further purification was not performed due to low yields. The recombinant proteins’ sperm-binding activity was investigated by immobilizing them in plastic wells in a solid-supported form [13]. We tested various protein amounts to determine the appropriate amount to saturate the adsorption to wells. We found that 0.8 μg of the protein was sufficient for saturation (Figure 2C). The number of bovine sperm bound to the bZP4 (136–464)-coated wells was significantly lower than that of bZP4 (25–464) and was around 50% of that of bZP4 (25–464) (Figure 2D), as shown previously [13]. Contrastingly, the number of bovine sperm bound to the pZP4 (137–466)-coated wells was nearly undetectable (Figure 2D), indicating that the sperm-binding activity of bZP4 (136–464) is species-selective.

Based on the low bovine sperm-binding activity for pZP4 (137–466), we systematically replaced the 290 to 340 region from the bovine amino acid sequence with the porcine amino acid sequence (Figure 2A). In pZP4 (333), Thr-332 of bZP4 (136–464) was replaced by the corresponding Asn at 333 of pZP4. In pZP4 (326–329), Lys-325 and Arg-328 of bZP4 (136–464) were replaced by the corresponding Glu at 326 and Gly at 329, respectively, of pZP4. In pZP4 (321), Lys-320 of bZP4 (136–464) was replaced by the corresponding Gln at 321 of pZP4. In pZP4 (312–315), Gln-311 and Asn-314 of bZP4 (136–464) were replaced by the corresponding His at 312 and Pro at 315, respectively, of pZP4. In pZP4 (299–302), Val-298 and Leu-301 of bZP4 (136–464) were replaced by the corresponding Ile at 299 and Phe at 302, respectively, of pZP4. These mutated fragments were expressed in Sf9 cells and partially purified (Figure 2B). For saturated adsorption on the plastic wells, 0.8 μg for each protein was enough (Figure 2C). The sperm-binding activity of bZP4 (136–464), containing mutations of pZP4 (333) and pZP4 (326–329) (bZP4 (136–464)/pZP4 (333, 326–329)), or pZP4 (333), pZP4 (326–329), and pZP4 (321) (bZP4 (136–464)/pZP4 (333, 326–329, 321)) (Figure 2D), did not differ significantly from that of bZP4 (136–464), suggesting that the bovine regions corresponding to these porcine amino acid regions, that is, bZP4 (332), bZP4 (325–328), and bZP4 (320), are not essential for bZP4 (136–464)’s sperm-binding activity. However, bZP4 (136–464)’s sperm-binding activity was significantly reduced when all five regions were mutated with the porcine sequences (bZP4 (136–464)/pZP4 (333, 326–329, 321, 312–315, 299–302)) (Figure 2D), indicating that the bovine regions bZP4 (311–314) and bZP4 (298–301) are required for its sperm-binding activity. This result was further confirmed with the mutation of pZP4 (312–315) and pZP4 (299–302) (bZP4 (136–464)/pZP4 (312–315, 299–302)) (Figure 2D). Interestingly, the sperm-binding activity of bZP4 (136–464) with the mutation of pZP4 (312–315) alone (bZP4 (136–464)/pZP4 (312–315)) was significantly reduced compared to that of bZP4 (136–464) (Figure 2D). Meanwhile, the sperm-binding activity of bZP4 (136–464)/pZP4 (312–315) was not significantly different from that of bZP4 (136–464)/pZP4 (312–315, 299–302). These results suggest that the corresponding bovine residues at 311 and 314 are necessary for the sperm-binding activity of bZP4 (136–464).

### 3.2. N-Glycosylation Site on bZP4 (290–340) Is Required for Sperm-Binding Activity

To investigate the specific residues that are involved in the species-selective sperm-binding activity, we focused on Gln-311 and Asn-314 of bZP4 (136–464). bZP4 (136–464) with mutation of Gln-311 to His (at 312 of pZP4) (bZP4 (136–464) Q311H) and bZP4 (136–464) with mutation of Asn-314 to Pro (at 315 of pZP4) (bZP4 (136–464) N314P) or Asp (bZP4 (136–464) N314D) (Figure 3A) were expressed in Sf9 cells and partially purified (Figure 3B). In the latter mutant, Asp, rather than Gln, was selected to imitate *N*-glycanase digestion at *N*-glycosylated Asn-314 [21]. We found that 0.8 μg for each protein is enough for saturation of adsorption on plastic wells (Figure 3C). We observed that the number of bovine sperm bound to the wells coated with bZP4 (136–464) N314P and bZP4 (136–464) N314D mutants was significantly lower than that of the bZP4 (136–464) wild-type (Figure 3D). Contrastingly, the number of bovine sperm bound to the wells coated with bZP4 (136–464) Q311H, a nearby mutant of the *N*-glycosylation site, was not significantly different from that of bZP4 (136–464) (Figure 3D). These results indicate that Asn-314 is predominantly involved in the sperm-binding activity of bZP4 (136–464).

bZP4 (136–464) has three putative *N*-glycosylation sites at Asn-202, Asn-219, and Asn-314 (Figure 1A) [25]. The *N*-glycosylation of Asn-314 from native bZP4 or recombinant bZP4 expressed in Sf9 cells has not been confirmed yet. *N*-linked carbohydrate chains of recombinant bZP4 expressed in Sf9 cells are pauci-mannose type chains, which are below 1000 Da [17]. bZP4 (136–464) and bZP4 (136–464) N314D were electrophoresed side by side on the same SDS-polyacrylamide gel to detect small difference in their mobilities on the gel (Figure 3E). The mobilities of bZP4 (136–464) and bZP4 (136–464) N314P were also compared in the same way. Both bZP4 (136–464) N314D and bZP4 (136–464) N314P showed slightly higher mobilities than that of bZP4 (136–464). This result indicates that Asn-314 is potentially *N*-glycosylated, and mutation of this residue removes one *N*-glycan, slightly increasing its mobility on the SDS gel. These results suggest that *N*-glycosylated Asn-314 is involved in the bovine sperm-binding activity of bZP4 (136–464).

### 3.3. N-Glycosylation Sites on Both bZP3 and bZP4 Are Involved in Sperm-Binding Activity

Recombinant bZP3 and bZP4 expressed in Sf9 cells form a heterocomplex that exhibits bovine sperm-binding activity [17]. Moreover, the complex between the *N*-terminal fragment of bZP3 (32–178) (Figure 1C and Figure 4A) and bZP4 inhibits sperm–ZP binding, as shown using competitive inhibition assays. The inhibitory activity requires *N*-glycosylation at Asn-146 of bZP3 (32–178) [20]. In this study, we analyzed the involvement of the *N*-glycosylation site at Asn-314 of bZP4 (136–464) in sperm-binding activity in the context of bZP3 (32–178)/bZP4 (136–464) complex using the solid support assay.

Since bZP4 (136–464) shows independent sperm-binding activity [13], it was *N*-terminally FLAG-tagged instead of His-tagged to remove the free bZP4(136–464) at the purification step of bZP3 (32–178)/bZP4 (136–464) complex using the TALON resin. His-tagged bZP3 (32–178) and FLAG-tagged bZP4 (136–464) were co-expressed in Sf9 cells and recovered as a mixture of bZP3 (32–178)/bZP4 (136–464) complex and free bZP3 (32–178) from the culture supernatants (Figure 4B). The number of bovine sperm bound to wells coated with bZP3 (32–178)/bZP4 (136–464) was around 40% lower than that observed in bZP4 (25–464)-coated wells, a 100% binding activity control (Figure 4D). This is consistent with our previous result showing that bZP4 (136–464) alone has reduced sperm-binding activity [13].

Next, we investigated the involvement of the *N*-glycosylation on bZP3 (32–178)/bZP4 (136–464) by mutating the *N*-glycosylation sites either at Asn-146 on bZP3 (32–178) or at Asn-314 on bZP4 (136–464) or both sites to Asp (Figure 4A). The resulting mutants, bZP3 (32–178) N146D/bZP4 (136–464), bZP3 (32–178)/bZP4 (136–464) N314D, and bZP3 (32–178) N146D/bZP4 (136–464) N314D, were expressed in Sf9 cells and recovered from culture supernatants using a His-tag attached to bZP3 (32–178) or a bZP3 (32–178) N146D fragment (Figure 4B). These ZP proteins were contaminated with the proteins around 32 kDa, but further purification was not performed due to their low yields.

We found that 0.8 μg of bZP3 (32–178) N146D/bZP4 (136–464), bZP3 (32–178)/bZP4 (136–464) N314D, and bZP3 (32–178) N146D/bZP4 (136–464) N314D was sufficient for saturation of the adsorption on plastic wells, similar to that of bZP3 (32–178)/bZP4 (136–464) (Figure 4C).

The number of bovine sperm bound to wells coated with bZP3 (32–178) N146D/bZP4 (136–464) was significantly lower compared to that bound to bZP3 (32–178)/bZP4 (136–464)-coated wells (Figure 4D). This was consistent with a previous finding that the mutation of Asn-146 to Asp on bZP3 (32–178) reduces the sperm-binding activity of bZP3 (32–178)/bZP4 (25–464) complex [20]. The sperm-binding activity of bZP3 (32–178)/bZP4 (136–464) N314D was significantly reduced compared to bZP3 (32–178)/bZP4 (136–464). This suggests that mutating Asn-146 to Asp on bZP3 (32–178) possibly has additional effects on the sperm-binding activity of bZP3 (32–178)/bZP4 (136–464) N314D complex (Figure 4D). However, there were no significant differences in sperm-binding activities between any pair among the three complexes, bZP3 (32–178) N146D/bZP4 (136–464), bZP3 (32–178)/bZP4 (136–464) N314D, and bZP3 (32–178) N146D/bZP4 (136–464) N314D. These results suggest that both *N*-glycosylation sites of bZP3 (32–178) and bZP4 (136–464) are necessary for sperm-binding activity.

## 4. Discussion

The sperm-binding activity of bZP4 depends on its *N*-terminal ZP-N1 domain and the region between residues 290 and 340, with the former showing higher sperm-binding activity than the latter [13]. bZP4 has four potential *N*-glycosylation sites at Asn-71, -202, -219, and -314 [25]. We found that the potential *N*-glycosylation site at Asn-71 of the *N*-terminal ZP-N1 domain was not involved in sperm-binding activity, as mutation of the site did not change the sperm-binding activity of this domain [19]. In this study, we further examined the sperm-binding site of the residues 290–340 of bZP4. We found that recombinant pZP4 (137–466) showed very low binding activity toward bovine sperm (Figure 2D), even though only eight amino acid residues in the 291–341 region of pZP4 are different from those at corresponding sites in the 290–340 region of bZP4 (Figure 2A). Therefore, our strategy for identification of sperm-binding sites in the 290–340 region of bZP4 was to systematically replace the 290–340 region from the bovine amino acid sequence with the porcine amino acid sequence and to examine bovine sperm-binding activities of the bZP4 mutants. As a result, we found that the *N*-glycosylation site at Asn-314 of bZP4 is important for its sperm-binding activity. We do not know, when each of the eight amino acid residues is mutated to other amino acids, such as Ala, whether the mutation would affect the sperm-binding activity of the 290–340 region of bZP4. This remains to be clarified.

Our competitive inhibition assays showed that neither bZP3 nor bZP4 expressed in Sf9 cells inhibited the binding of bovine sperm to ZP [17], indicating that individually, either bZP3 or bZP4 in a solution does not have sperm-binding activity. However, bovine sperm binds to bZP4-coated plastic wells but not to bZP3-coated ones [13], suggesting that in a solid-supported multivalent form, bZP4 shows sperm-binding activity, but bZP3 does not. The competitive inhibition assays also showed that the complex between the *N*-terminal fragment of bZP3 ranging from 32 to 178 and bZP4 recombinantly expressed in Sf9 cells inhibited sperm binding to ZP [20]. The mutation of the *N*-glycosylation site at Asn-146 of the bZP3 fragment to Asp significantly reduces the inhibitory activity of the complex between the bZP3 fragment and bZP4, but the mutation does not reduce the interaction between the bZP3 fragment and bZP4 [20]. This indicated that when complexed with bZP4, the *N*-glycosylated Asn-146 of bZP3 is probably involved in sperm-binding activity. Here, we found that mutation of Asn-314 of bZP4 to Asp significantly reduced the sperm-binding activity of the complex between the bZP3 fragment and the bZP4 fragment lacking the *N*-terminal ZP-N1 domain. Taken together with the previous results, the presence of both *N*-glycosylated Asn-146 of bZP3 and *N*-glycosylated Asn-314of bZP4 is essential for the formation of an active sperm-binding complex.

When expressed in Sf9 cells, the recombinant bZP4 contains pauci-mannose type *N*-glycans with α-Man residues at the non-reducing termini [17]. Bovine sperm exhibits a preference for α-Man residues, as shown by its binding to plastic wells coated with α-mannosylated glycolipid analog [26]. The binding of bovine sperm to native ZP requires non-reducing terminal α-Man residues [16]. Therefore, these residues of the recombinant bZP3/bZP4 complex might participate in the binding of bovine sperm to the complex. However, it remains to be clarified whether the *N*-glycans directly bind to bovine sperm, or whether they have roles in correct folding of the polypeptide moiety of the sperm-binding site.

Native pZP4 purified from ovaries lacks the *N*-terminal ZP-N1 domain [27,28], which could be due to post-translational processing, resulting in a mature pZP4 polypeptide that stretches from Asp-137 to Arg-466. pZP4 has three *N*-glycosylation sites at Asn-203, Asn-220, and Asn-333. Mutation of Asn-203 or Asn-220 to Asp significantly reduces the sperm-binding activity of the pZP3/pZP4 complex, whereas that of Asn-333 to Asp does not [21]. Based on the 3D model of pZP4, Asn-203 and Asn-220 are in close proximity [3,29,30]. A 3D model of pig ZP filament was proposed based on the cryo-EM structure of human uromodulin filament [29,30]. In this model, the *N*-glycosylation site at Asn-220 of the pZP4-ZP-N domain, *O*-glycosylation sites in the hinge region of pZP3, Thr-155, Thr-161, and Thr-162, and the *N*-glycosylation site at Asn-271 of pZP3 were located close to the interface of the pZP3-ZP-C and pZP4-ZP-N domains that form a sperm-binding surface. This region might also contain the *N*-glycosylated Asn-203, which is close to Asn-220 of the pZP4-ZP-N domain. However, the involvement of the *N*-glycosylation site at Asn-271 of pZP3 in the sperm-binding activity of the pZP3/pZP4 complex has not been clarified yet using in vitro sperm-binding assays. In chicken, *O*-glycosylation at Thr-168 in the hinge region of ZP3 homolog was shown to be important for the sperm-binding activity of ZP3 homolog [9], and this glycosylation site is located in the interfaces of domains, as proposed based on the cryo-EM structure of human uromodulin filament [29].

**Figure 5:**
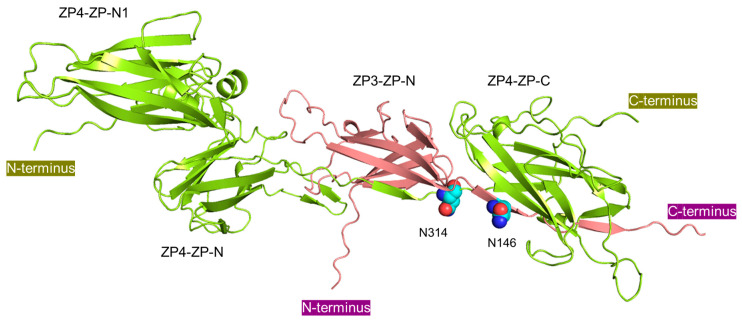
A predicted structural model of the bZP3 (32–178)/bZP4 (25–464) protein complex. The model is a top-ranked model made using AlphaFold2. bZP3 (32–178) and bZP4 (25–464) are shown in purple and green, respectively. The side chains of Asn-146 and Asn-314, which are the proposed sperm-binding sites on bZP3 (32–178) and on bZP4 (136–464), respectively, are indicated by the balls. These two *N*-glycosylated residues are located near the interface between the bZP3 ZP-N and bZP4 ZP-C domains.

The 3D model of the bZP3 fragment/bZP4 complex, proposed by AlphaFold2/ColabFold [24], showed that Asn-146 of bZP3 and Asn-314 of bZP4 are in close proximity, potentially forming a sperm recognition site at the interface of the bZP3-ZP-N and bZP4-ZP-C domains (Figure 5). Asn-314 is located at the *N*-terminal edge of the ZP-C domain within a consensus sequence called the internal hydrophobic patch (IHP) [8]. In the IHP, the amino acid at this position is usually Pro, as shown in the pZP4 sequence (Figure 2A). The Asn at this position in ZP4 might be unique to bovines, making the *N*-glycosylation at this site a unique feature of bZP4. Therefore, the formation of a sperm-binding site including *N*-glycosylated Asn residues at the ZP3-ZP-N-ZP4-ZP-C domain interface might also be unique to bovines.

In our previous study, incubation of bovine sperm with solubilized native bovine ZP for 3 h in BO solution, the same medium as used in the present study, induced only a 5% acrosome reaction [17]. Therefore, we suppose that in the present study using bZP protein-coated plastic wells, the acrosome reaction of bovine sperm was not induced.

## 5. Conclusions

This study and the previous studies reporting on bovine sperm-binding sites on the bZP3/bZP4 complex, all of which were performed with in vitro systems, suggest that bovine acrosome-intact sperm bind to the *N*-terminal ZP-N1 domain of bZP4 and to the interface between the bZP3-ZP-N and bZP4-ZP-C domains. Future studies should investigate the sperm factors that recognize these sites to show whether these sperm-binding sites have physiological roles in in vivo fertilization.

## Data Availability

Raw data are available on request from the corresponding author (N.Y.).

## Supplementary Materials

The following supporting information can be downloaded at: https://www.mdpi.com/article/10.3390/biom13111636/s1.
