# Supplementary material for: N-Glycosylation Site in the Middle Region Is Involved in the Sperm-Binding Activity of Bovine Zona Pellucida Glycoproteins ZP3 and ZP4
Source: Biomolecules. 2023 Nov 10;13(11):1636. doi: 10.3390/biom13111636 (PMC10669178; doi:10.3390/biom13111636)
Supplement: Supplementary file 1 [file biomolecules-13-01636-s001.zip › biomolecules-2658895-supplementary.pdf]

Figure 2B

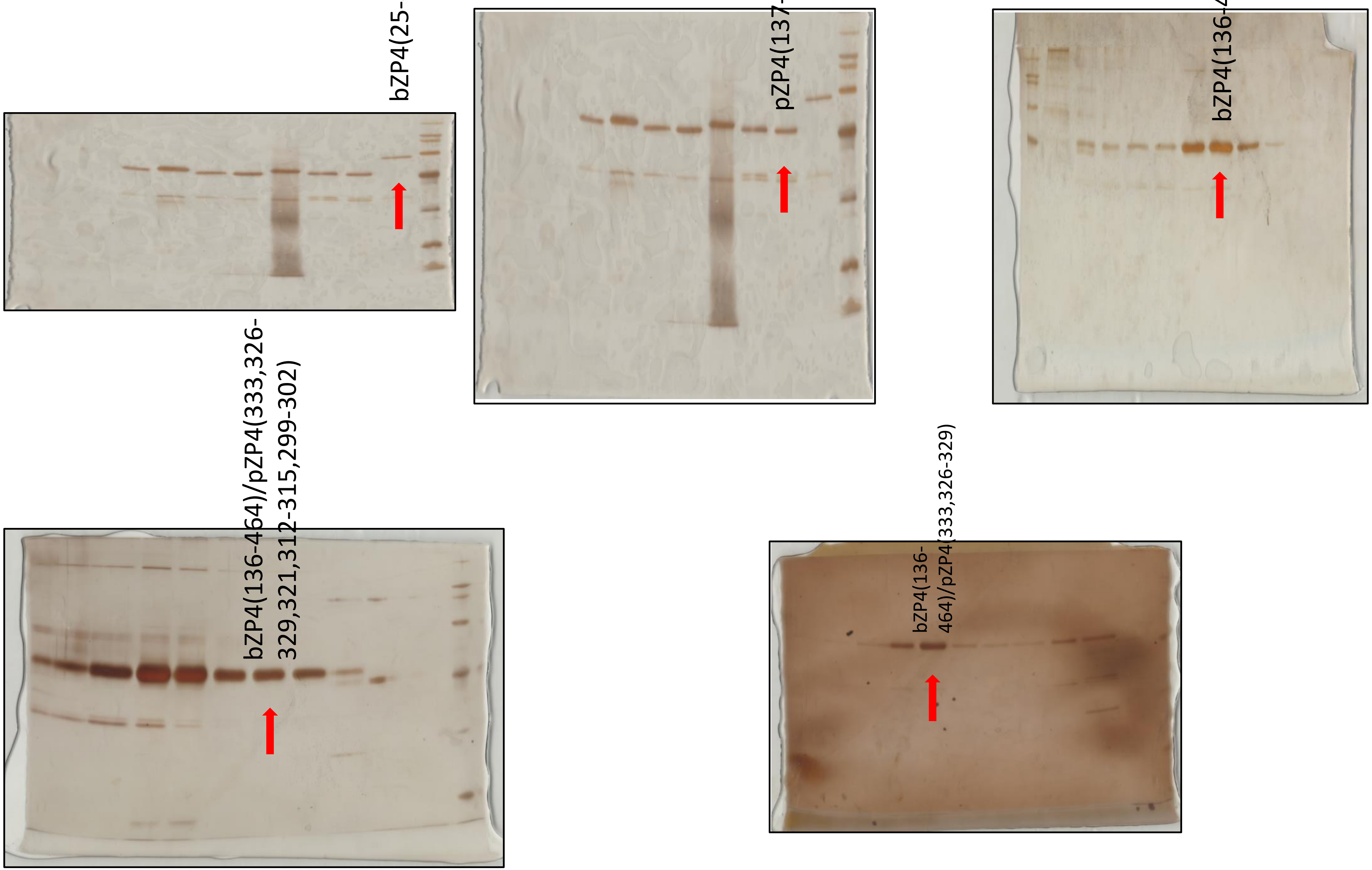

bZP4(25-464)
pZP4(137-462)
bZP4(136-464)
bZP4(136-464)/pZP4(333,326-329,321,312-315,299-302)
bZP4(136-464)/pZP4(333,326-329)

Figure 2B

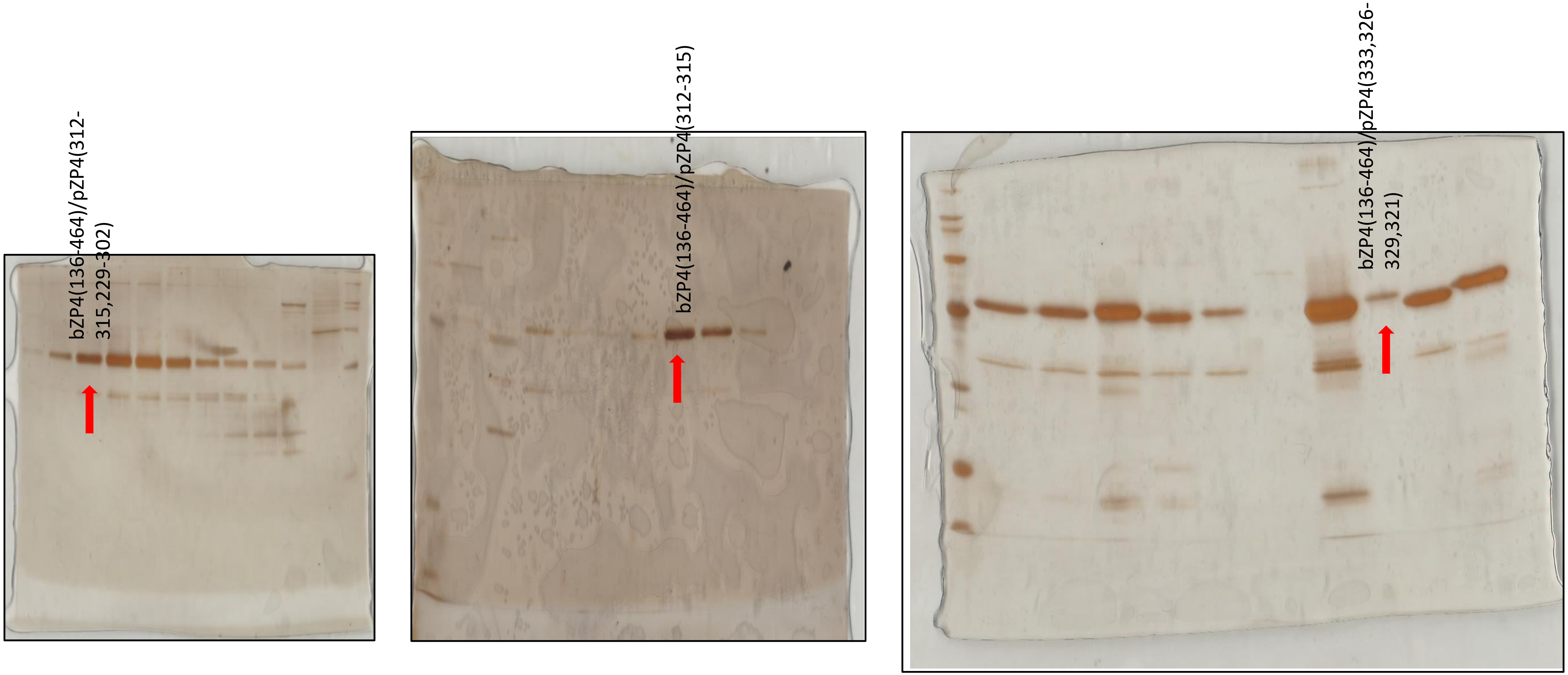

bZP4(136-464)/pZP4(312-315,229-302)
bZP4(136-464)/pZP4(312-315)
bZP4(136-464)/pZP4(333,326-329,321)

Figure 3B

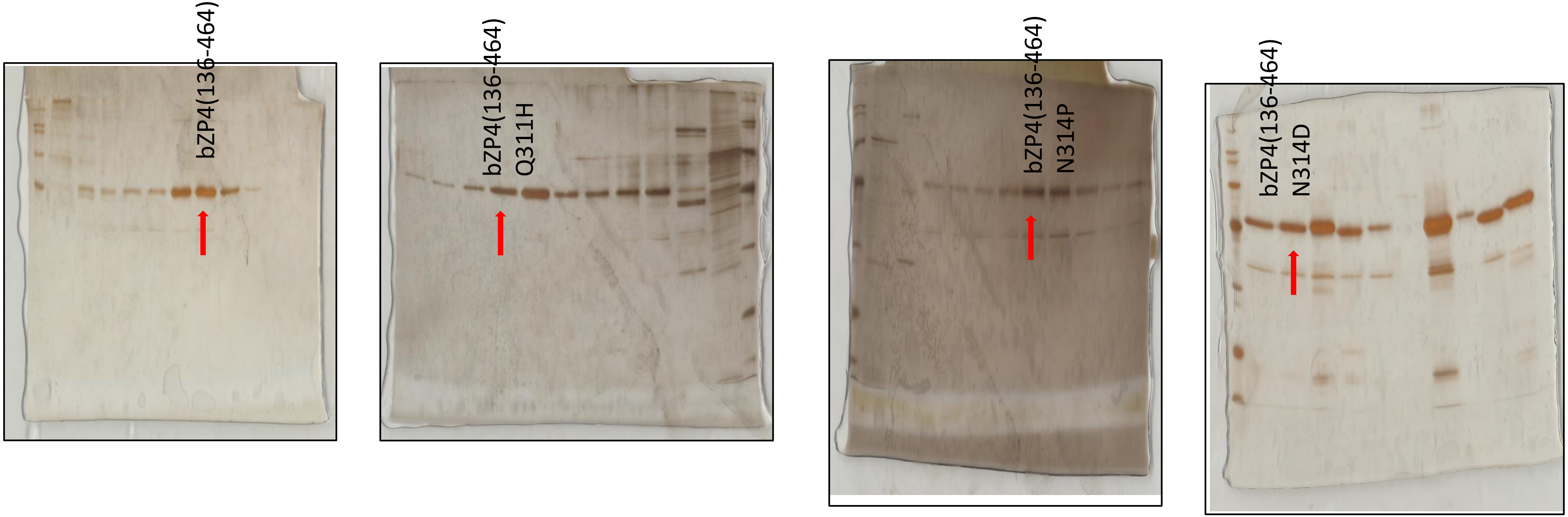
bZP4(136-464)
bZP4(136-464)
Q311H
bZP4(136-464)
N314P
bZP4(136-464)
N314D

Figure 3E

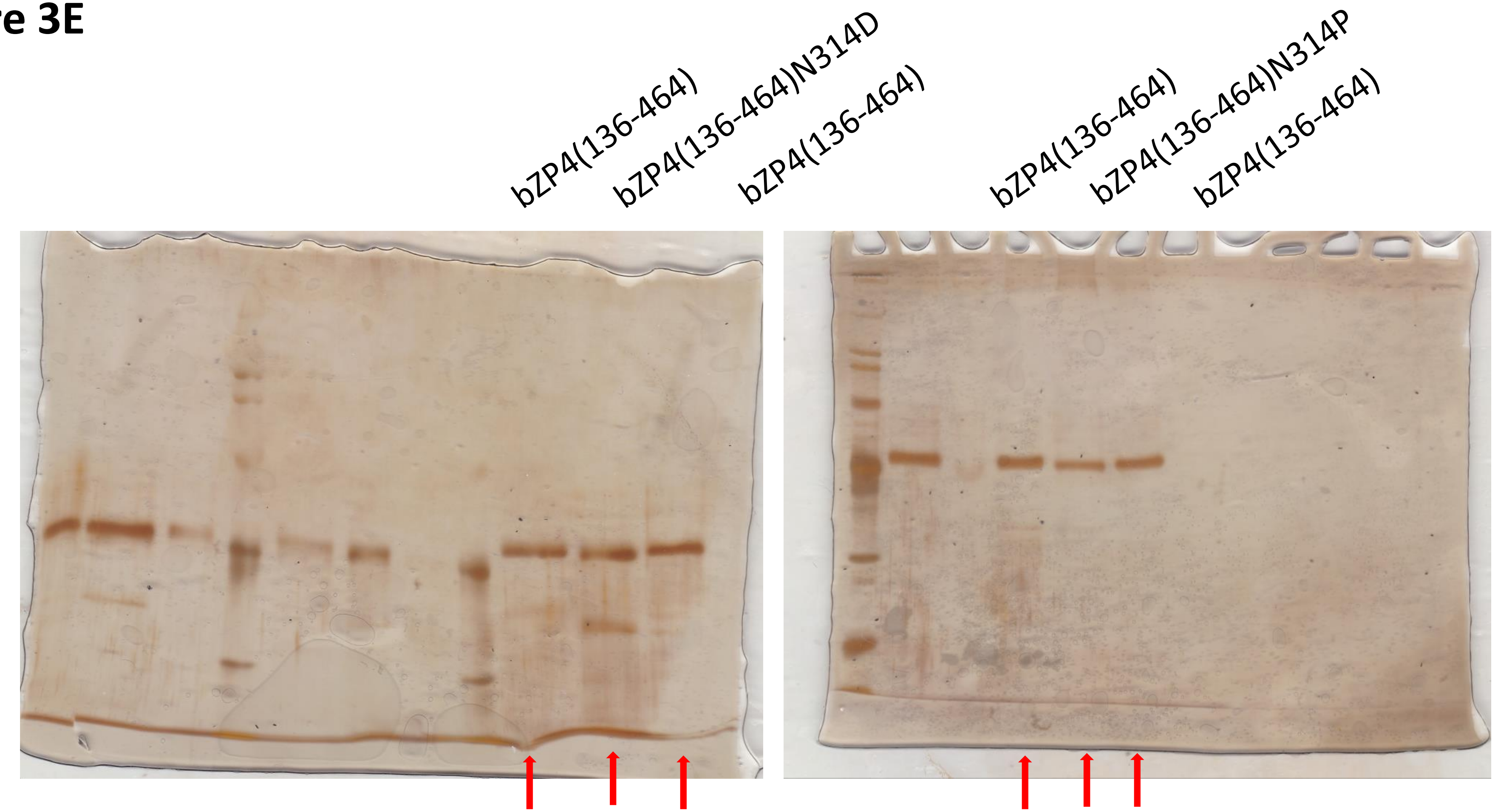
bZP4(136-464)
bZP4(136-464)N314D
bZP4(136-464)
bZP4(136-464)
bZP4(136-464)N314P
bZP4(136-464)

Figure 4B

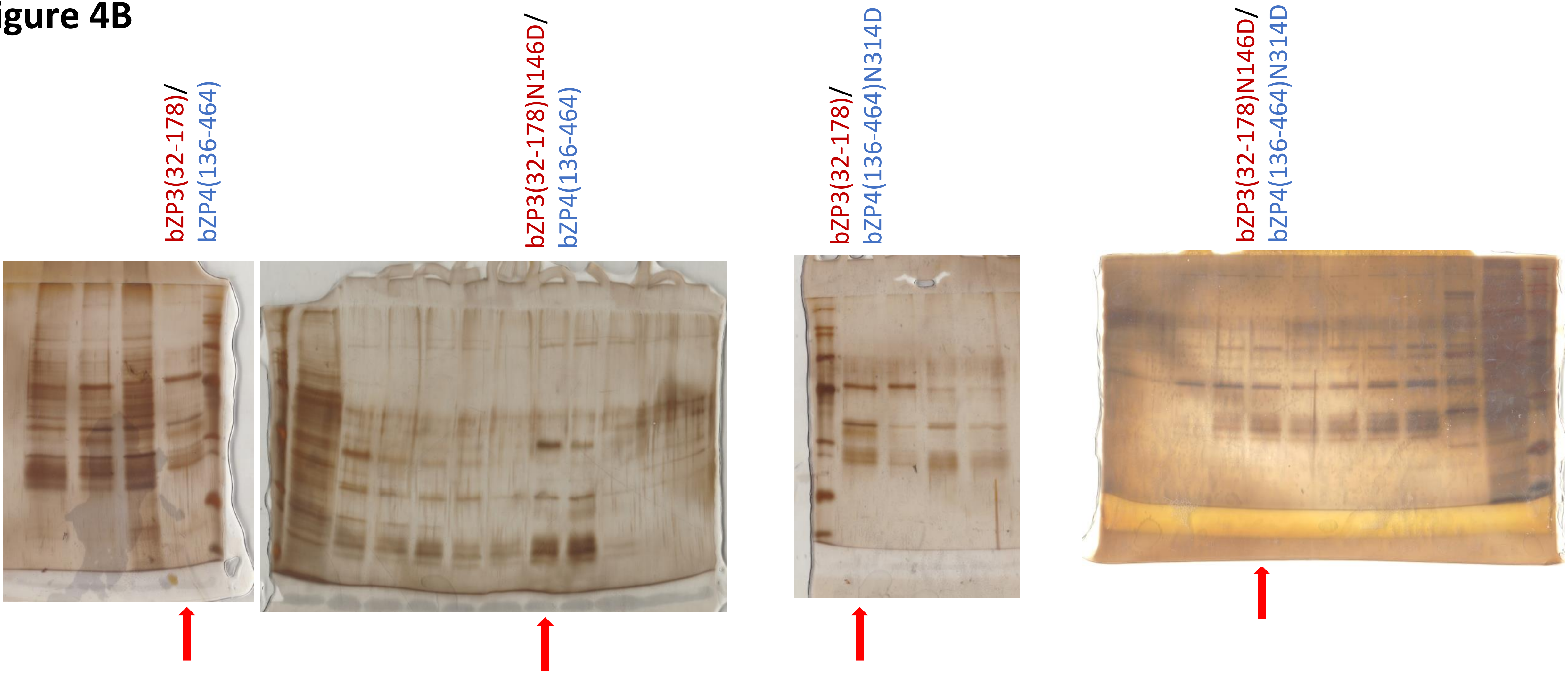
bZP3(32-178)/
bZP4(136-464)
bZP3(32-178)N146D/
bZP4(136-464)
bZP3(32-178)/
bZP4(136-464)N314D
bZP3(32-178)N146D/
bZP4(136-464)N314D
